# Randomized double blind placebo-controlled study to demonstrate that antibiotics are not needed in moderate acute exacerbations of COPD – The ABACOPD Study

**DOI:** 10.1186/1471-2466-15-5

**Published:** 2015-01-27

**Authors:** Gernot G U Rohde, Armin Koch, Tobias Welte, for the ABACOPD study group

**Affiliations:** Department of Respiratory Medicine, Maastricht University Medical Centre, Maastricht, the Netherlands; Institute for biometry, Hannover Medical School (MHH), Hannover, Germany; Department of Respiratory Medicine, Hannover Medical School (MHH), Hannover, Germany; CAPNETZ Stiftung, Hannover, Germany; Biomedical Research in Endstage and Obstructive Lung Disease Hannover (BREATH), Member of the German Center for Lung Research (DZL), Hannover, Germany

## Abstract

**Background:**

Antibiotic-resistant strains of pathogenic bacteria are increasingly prevalent in hospitals and the community. Acute exacerbations of COPD (AE-COPD) often result in administration of antibiotics although more than half of exacerbations are associated with detection of respiratory viruses and potentially pathogenic bacteria can only be detected in 20-30% of cases. There is a paucity of placebo-controlled clinical trials and up to today no single study has been powered sufficiently to prove the efficacy of antibiotic treatment in AE-COPD. Most studies so far did not include current standards of care comprising administration of systemic corticosteroids.

**Methods/Design:**

A total of 980 patients with moderate acute exacerbations will be included in 22 German centers (hospitals and private practices). Patients will receive a standardized treatment for exacerbation including systemic corticosteroids, inhaled bronchodilators and supplementary oxygen if needed and will be randomized to additional treatment with placebo or antibiotic (oral sultamicillin) for five days.

The primary endpoint is clinical failure defined by need for additional antibiotic treatment until day 30. Secondary endpoints will assure that management of AE-COPD without antibiotics does not result either in increased occurrence of relapse, new exacerbations, prolonged recovery, or unwanted long-term consequences.

**Discussion:**

ABACOPD will be the first sufficiently powered double-blind placebo-controlled study in the field to systematically assess the question whether antibiotics, known to increase antibiotic resistance, are really needed in a well-defined patient cohort receiving state-of-the art treatment in all other aspects.

**Trial registration number:**

ClinicalTrials.gov: NCT01892488.

## Background

Antibiotic resistance is increasing in hospitals as well as in the community. It has been shown that antibiotic prescriptions without a clear indication contribute significantly to this increase [1]. Acute exacerbations of COPD (AE-COPD) represent a frequent clinical situation in which administration of antibiotics is considered. In 2010 nearly 700.000 hospital discharges for COPD in the U.S. [2] have been registered. A retrospective cohort study identified that antibiotics were prescribed in 85% of 69 820 patients admitted for acute exacerbations of COPD to 360 hospitals throughout the United States [3]. This is impressive as evidence of efficacy of antibiotic treatment of AE-COPD is scarce. It is estimated that about 70% of exacerbations are infectious in origin [4]. However, only 20-30% are associated with detection of bacteria by sputum culture [4]. Importantly, detection of pathogens by culture is not per se a proof of infection. Particularly in COPD patients growth of bacteria in sputum cultures can also result from colonization of the airways [5]. Up to now colonization is not considered as a clear indication for antibiotic treatment. In addition, others and we have shown that respiratory viruses are detected in more than 50% of exacerbations and also have to be considered as major infectious triggers [6, 7]. In viral infections antibiotic therapy is not expected to be effective [8]. Moreover antibiotic treatment is associated with increased morbidity, mainly reflected by gastro-intestinal complications such as diarrhoea, often caused by C. difficile [9].

A recent Cochrane review assessed the effects of antibiotics in the management of acute COPD exacerbations on treatment failure as observed between seven days and one month after treatment initiation (primary outcome) and on other patient-important outcomes (mortality, adverse events, length of hospital stay) [10]. In this most recent systematic review using GRADE methodology [11] 16 trials with 2068 participants were included. Outpatients were considered to have mild to moderate exacerbations. Only the most recent trial showed a benefit of antibiotic treatment and drove the results towards beneficial effects of antibiotics [12]. When the meta-analysis was restricted to currently available drugs however, there was no significant benefit but just a trend in favour of antibiotics (RR 0.80; 95% CI 0.63 to 1.01; I2 = 33%) [10]. However, evidence of high quality showed that antibiotics significantly reduced the risk of treatment failure in inpatients with severe exacerbations (ICU not included) (RR 0.77; 95% CI 0.65 to 0.91; I2 = 47%) regardless of whether restricted to current drugs or not. There still is only one trial with 93 patients admitted to the ICU, which was also analysed in this review [13]. This trial showed a large and statistically significant effect on treatment failure (RR 0.19; 95% CI 0.08 to 0.45; high-quality evidence) [10]. In studies addressing mortality evidence of low-quality from four trials in inpatients showed virtuality no effect of antibiotics on mortality (Peto OR 1.02; 95% CI 0.37 to 2.79) [10]. Length of hospital stay (in days) was similar in the antibiotics and placebo groups except for the ICU study. The overall incidence of adverse events was higher in the antibiotics groups (Peto OR 1.53; 95% CI 1.03 to 2.27). Patients treated with antibiotics experienced more diarrhoea based on three trials (Peto OR 2.62; 95% CI 1.11 to 6.17; high-quality evidence) [10]. The authors concluded on the basis of these findings that for outpatients and inpatients the results were inconsistent. The risk for treatment failure was significantly reduced in both inpatients and outpatients when all trials (1957 to 2012) were included but not when the analysis for outpatients was restricted to currently used antibiotics. Also, antibiotics had no statistically significant effect on mortality and length of hospital stay in inpatients and almost no data on patient-reported outcomes exist [10]. The appraisal of the results of this systematic review is largely impaired by the fact that there was no pre-defined standard treatment of AE-COPD including corticosteroids, even not in the most recent trials. Therefore it is impossible to judge the benefit of antibiotic treatment in addition to standard treatment of AE-COPD.

It is remarkable that on this brittle basis, antibiotic treatment for AE-COPD is recommended for patients with severe exacerbations or severe underlying COPD [8]. Moreover, most AE-COPD are mild to moderate and not severe and it seems difficult to extrapolate the findings from one positive ICU study to the less severe situations.

In conclusion there still is no clear recommendation for antibiotic treatment for these patients. These facts underline that a randomized, placebo-controlled trial on antibiotics for mild to moderate AE-COPD is warranted [8]. In light of the fact that respiratory viruses are detected in 50% of exacerbations a major task is the reduction of unnecessary antibiotic treatment in patients with mild to moderate COPD exacerbations to stop the threatening increase in AB resistance [8]. It has been convincingly shown that prudent reduction of antibiotic use results in a decrease of bacterial resistance without an increase in complications [14]. Taken this important aspect into account it seems that the risk-benefit ratio of antibiotic treatment for mild to moderate AE-COPD is negative and should therefore be discouraged. The most recent study [15] showed that it is safe to treat AE-COPD patients with all grades of disease severity (GOLD 1 to 4) with placebo if standardized treatment is provided. All studies performed so far were designed to prove efficacy of antibiotics in AE-COPD [8].

Against this background we have designed a randomized double blind placebo-controlled study to demonstrate that antibiotics are not needed in moderate acute exacerbations of COPD – The ABACOPD Study. ABACOPD is the first study designed to show non-inferiority of placebo in AE-COPD proving that antibiotics are not needed. This might have important impact to individual patients as well as to society. It has been shown in several trials that adverse events are more frequent in patients treated with antibiotics than in patients on placebo [16]. Hence the individual patient might benefit from reduced adverse events associated with antibiotic treatment if antibiotics are no longer prescribed for this indication. In addition the individual patient might also benefit from decreased selection of antibiotic resistant strains by reduction of antibiotic consumption. Society will clearly benefit if unnecessary antibiotic prescriptions will be reduced. This has been convincingly shown in other settings [14]. At last there is a potential economic impact. Antibiotic therapy can be costly and it has been shown that reduction in antibiotic use significantly reduces costs [17].

## Methods

### Objective

The ultimate goal is to reduce unnecessary antibiotic prescriptions, which drive the development of antibiotic resistance in the community. The primary objective of ABACOPD is to demonstrate in a sufficiently sized clinical study that there is no relevant increase in the “failure-rate” for patients with acute moderate exacerbations of COPD (AE-COPD) treated with placebo instead of antibiotic treatment both on top of standard of care. A patient is classified as treatment failure if additional antibiotic therapy is required during the initial treatment period or until the test of cure visit (TOC at day 30, primary endpoint). Secondary objectives are to demonstrate that patients on placebo do not experience more frequent relapse or show prolonged symptoms. A patient is classified as relapse if new antibiotic therapy for AE-COPD is required within the first six months after TOC. Clinical cure rates at end of treatment (EOT) and TOC will inform about duration of symptoms and speed of recovery.

### Intervention

Each patient will receive inhaled short-acting bronchodilators, systemic corticosteroids (prednisolone 40 mg/d p.o. for 7–10 days) and oxygen in case of hypoxemia in a standardized fashion. The pre-existing COPD medication will be continued as indicated. All patients in the study will receive, per randomized allocation, treatment with either antibiotic therapy or placebo. Those randomized to antibiotic therapy will receive 750 mg sultamicillin (two 375 mg capsules) twice a day (BID) for 5 days. An Aminopenicillin plus betalactamase inhibitor is the recommended antibiotic treatment for moderate AE-COPD in Germany [18]. The treatment duration of 5 days is chosen because the latest interventional trial in lower respiratory tract infections showed for patients with AE-COPD that 5 days of treatment is safe and effective and that even shorter duration can be used if procalcitonin guidance is employed [19]. Those patients randomized to the placebo will receive two placebo capsules twice a day (BID) for 5 days. The duration of treatment with study medications will be 5 days.

### Key inclusion criteria

Patients will be enrolled into the study when diagnosed with moderate acute exacerbation of COPD according to the definition of the Global initiative for chronic Obstructive Lung Disease (GOLD) without respiratory failure or need for intermediate or intensive care if they fulfill the following criteria:

- Male and non-pregnant female adults, older than 40 years of age, diagnosed with COPD stages I-IV as defined by GOLD
- Doctor’s diagnosis of acute (onset < 7 days) moderate exacerbation of COPD (definition see 3.2), necessitating the administration of systemic corticosteroids
- Procalcitonin level < 0.25 ng/mL (to rule out community acquired pneumonia or lower respiratory tract infection with a clear indication for antibiotic treatment)
- Smoking history of 10 Pack Years or more

### Key exclusion criteria

- Severe exacerbation defined by need for ventilatory support indicated by PaCO2 > 45 mmHg and/or pH < 7.35, mental confusion, or unstable comorbiditiy
- Pneumonia (proven by chest x-ray performed in suspected cases)
- Fever (>38.5°C)
- Known impaired hepatic or renal function
- Active or suspected tuberculosis infection of the respiratory tract
- Acute exacerbation of asthma
- Suspected or known hypersensitivity to, or suspected serious adverse reaction to Sultamicillin
- Immunosuppression or Immunosuppressive therapy
- Antibiotic use within 30 days prior to randomization
- An antibiotic is clearly indicated for treatment of a known infection
- Known MRSA colonization or infection
- Patients with known bronchiectasis

### Outcomes

#### Primary efficacy endpoint

A test of cure (TOC)-visit will be performed at day 30 after start of the treatment phase. A patient is classified as a treatment failure if additional antibiotic therapy is required during treatment period or until the TOC-visit. For each treatment arm a failure rate will be computed.

### Key secondary endpoint(s)

- Relapse rate at late follow-up (LFU-1) 6 months after the start of treatment, and time to relapse; exacerbation rate at late follow-up (LFU-2, one year after start of treatment), and time to next exacerbation; clinical cure rate at the EOT visit and clinical cure rate at the TOC visit (both determined by patient-reported outcome (diary cards). Spirometry will be assessed at LFU-2 to assess long-term consequences.

Assessment of safety:

### Key criteria for safety are

- Adverse events
- Serious adverse events
- Incidence of complications (Need for intensive care unit treatment, need for ventilatory support)
- Vital status

### Statistical analysis

#### Efficacy/test accuracy

A patient is classified as a treatment failure if additional antibiotic therapy is required during treatment period or until the test of cure visit (TOC at day 30, primary endpoint).

The primary analysis will be stratified for centre. A two-sided 95%-confidence interval will be computed for the difference in failure rates for placebo minus experimental treatment group. The null-hypothesis of relevant inferiority of the placebo treatment will be rejected in favor of the alternative that antibiotic treatment is not required in this patient population, if the upper bound of the confidence interval does not exceed 10%, which is standard for the comparison of antibiotic treatments. The primary analysis will be conducted on the ITT-population including all randomized patients that have taken experimental drug or placebo at least once. A conservative rule will count missing values in the placebo-group with treatment failure, and as success in the active treatment group.

### Sample size calculation

A limited number of studies have compared antibiotic treatment to placebo in patients with AE-COPD. Jorgensen et al. [20] found in 1992 a 63% success rate with placebo as compared to 64% with Amoxicillin after 7 days of treatment of uncomplicated exacerbations of chronic bronchitis. In contrast Anthonisen et al. [21] found in 1987 a 55% success rate with placebo and a 68% response rate with antibiotic treatment for 10 days. Daniels et al. [15] observed in 2010 a clinical success in the first study using a standardized treatment including systemic corticosteroids in 61% of patients from the doxycycline group and 53% from the placebo group. In all these studies background standard of care has not been optimized and it is assumed that this is the reason for observed differences in small studies. It is thus assumed that the response rate in the study population will be in the order of 65%. With a one-sided type 1 error of 2.5% 479 patients per group have to be recruited to reject the null-hypothesis of relevant inferiority of placebo treatment with 90% power and a non-inferiority margin of 10%. (Sample-size calculation has been performed with nQuery-Advisor 7.0).

### Safety

Adverse events and serious adverse events will be summarized per treatment group and descriptively compared between treatment-groups with a Chi-Square test.

Secondary endpoints:

Secondary analyses will investigate the relapse rate (new antibiotic treatment needed for AE-COPD within six months), time to relapse, the treatment effect with respect to clinical cure rates and failure rates at end-of-treatment (EOT)-visit and the TOC-visit in the modified ITT (mITT) population and the clinical evaluable (CE)-population. Exacerbation rates and time to next exacerbation will be assessed within 1 year after the index exacerbation.

### Study populations

We have calculated that 1300 patients have to be assessed for eligibility to have 980 patients to be allocated to trial. Ultimately 960 patients will be analysed. The total duration of the trial is calculated to be 36 months. Based on the feedback of the study centres of our research network we have calculated that 22 German centers need to be involved to guarantee adequate recruitment.

### Consent statement

Written informed consent for participation in the study will be obtained from all participants.

### Ethics statement

The study has been approved by the ethics committee (EC) of the Hannover Medical School (Nr. 6414 M) and the ECs of all local clinical centers.

## Discussion

The novel aspect of the proposed trial is to investigate non-inferiority of placebo against adequate antibiotic treatment in addition to well-defined standardized treatment of moderate AE-COPD in order to show that antibiotics are not needed for this indication. The generally accepted standard of care for patients with AE-COPD is administration of inhaled short-acting bronchodilators, systemic corticosteroids (prednisolone 40 mg/d for 7–10 days) and oxygen in case of hypoxemia. All patients in this study will receive this treatment during exacerbation. In the meantime a study has been published indicating that even shorter duration of corticosteroid treatment (5 days) is effective [22]. We consider this as important new information to the field but do not think that this has relevant implications for our study and the standard treatment is the same for both study arms. The role of antibiotics is debated and in the recent trial from Daniels et al. antibiotic treatment was not significantly more effective than placebo. Clinical success was 53% in the placebo group compared to 61% in the doxycycline group, p = 0.32 [15]. Llor et al. have very recently published a trial of amoxicillin/clavulanate (AMX) 500/125 mg three times a day compared to placebo three times a day for 8 days in patients with mild to moderate COPD [12]. 158 patients received AMX and 152 placebo. However, only 26 (16.5%) and 27 (17.8%) received oral corticosteroids, which again points to the problem of insufficient standard treatment and the difficulty to validly assess the effects of antibiotic treatment.

All other older trials are as well not applicable to this question as they did not use standardized treatment for AE-COPD including systemic corticosteroids. Therefore it is justified that patients will be randomised to either receive additional antibiotic treatment of or placebo if standard treatment (see above) is provided.

The choice of antibiotic treatment in AE-COPD is also a matter of debate. However, the most recent discussion clearly indicated that patients are likely to derive greatest benefit from early treatment with the most potent antibiotic therapy, such as amoxicillin/clavulanate and respiratory fluoroquinolones which have a broad spectrum of activity against likely pathogens [23]. Sultamicillin belongs to the first category and therefore we are confident that it is a good choice for this study.

If the primary study outcome is met the community will benefit from reduced consumption of antibiotics. The study will include 960 patients and will be sufficiently powered to precisely estimate a potential risk from waiving anti-biotic treatment and to definitively answer this crucial clinical question.

A limited number of studies have compared antibiotic treatment to placebo in patients with AE-COPD. Jorgensen et al. [20] found in 1992 a 63% success rate with placebo as compared to 64% with Amoxicillin after 7 days of treatment of uncomplicated exacerbations of chronic bronchitis. In contrast Anthonisen et al. [21] found in 1987 a 55% success rate with placebo and a 68% response rate with antibiotic treatment for 10 days. Daniels et al. [15] observed in 2010 a clinical success in the first study using a standardized treatment including systemic corticosteroids in 61% of patients from the doxycycline group and 53% from the placebo group. In all these studies background standard of care has not been optimized and it is assumed that this is the reason for observed differences in small studies. It is thus assumed that the response rate in the study population will be in the order of 65%. With a one-sided type 1 error of 2.5% 479 patients per group have to be recruited to reject the null-hypothesis of relevant inferiority of placebo treatment with 90% power and a non-inferiority margin of 10%. (Sample-size calculation has been performed with nQuery-Advisor 7.0).

The trial is designed and powered to prove that antibiotic therapy is not necessary in addition to standard of care in patients with moderate AE-COPD with the aim to reduce unnecessary antibiotic prescriptions and the ultimate goal to reduce antibiotic resistance. This is the first study in the field to use a non-inferiority trial design including a placebo arm. Other trials have used superiority design for antibiotics and yielded inconclusive results. These trials were all designed with the hypothesis that antibiotic therapy should be superior to placebo. In this trial we will investigate whether use of placebo is not inferior to antibiotic treatment and have chosen a tight non-inferiority margin to justify that antibiotic treatment should be withheld in this patient population in the future. This is clinically relevant for the target population as adverse effects and antibiotic resistance could be decreased if antibiotic treatment will be shown not be necessary in this trial.

The sample size of this trial is assumed to be sufficient to determine changes in relapse rates, time to relapse, clinical cure rates at EOT and TOC and to assess long-term consequences of sparing anti-biotic treatment.

This is a randomized and double-blind clinical trial with placebo matching tablets for active treatment. Block-randomization will be used. Randomization will be stratified for centre. All randomized patients that have received study medication at least once will be included into the primary intention-to-treat (ITT)-analysis. This is a non-inferiority trial and usually a per-protocol analysis would be used as primary analysis. However, the impact of potential missing values could be anti-conservative in the per-protocol analysis when demonstrating non-inferiority to placebo as well (pts with no improvement to placebo drop) and the ITT population is used here with a strategy that should maximize differences between treatment groups in case of uncertainty about outcome, because irrelevance of differences between treatment strategies is the objective to be demonstrated. In this complex situation missing values will be counted as failure in the placebo-group and as a success for patients in the antibiotics group. It is obvious that all attempts have to be undertaken to follow all patients to the end of the study.

In conclusion we believe that ABACOPD will be the first sufficiently powered double-blind placebo-controlled study in the field to systematically assess the question whether antibiotics, known to increase antibiotic resistance, are really needed in a well-defined patient cohort receiving state-of-the art treatment in all other aspects.
